# Restricted gene flow between resident *Oncorhynchus mykiss* and an admixed population of anadromous steelhead

**DOI:** 10.1002/ece3.3338

**Published:** 2017-09-08

**Authors:** Andrew P. Matala, Brady Allen, Shawn R. Narum, Elaine Harvey

**Affiliations:** ^1^ Columbia River Inter‐Tribal Fish Commission Hagerman Fish Culture Experiment Station Hagerman ID USA; ^2^ Columbia River Research Laboratory USGS‐Western Fisheries Research Center Cook WA USA; ^3^ Yakama Nation Fisheries Goldendale WA USA; ^4^ Present address: Bonneville Power Administration P.O. Box 3621, Portland OR 97208 USA

**Keywords:** admixture, Columbia River, habitat heterogeneity, immigration, resident redband trout, steelhead trout, sympatry

## Abstract

The species *Oncorhynchus mykiss* is characterized by a complex life history that presents a significant challenge for population monitoring and conservation management. Many factors contribute to genetic variation in *O. mykiss* populations, including sympatry among migratory phenotypes, habitat heterogeneity, hatchery introgression, and immigration (stray) rates. The relative influences of these and other factors are contingent on characteristics of the local environment. The Rock Creek subbasin in the middle Columbia River has no history of hatchery supplementation and no dams or artificial barriers. Limited intervention and minimal management have led to a dearth of information regarding the genetic distinctiveness of the extant *O. mykiss* population in Rock Creek and its tributaries. We used 192 SNP markers and collections sampled over a 5‐year period to evaluate the temporal and spatial genetic structures of *O. mykiss* between upper and lower watersheds of the Rock Creek subbasin. We investigated potential limits to gene flow within the lower watershed where the stream is fragmented by seasonally dry stretches of streambed, and between upper and lower watershed regions. We found minor genetic differentiation within the lower watershed occupied by anadromous steelhead (*F*_ST_ = 0.004), and evidence that immigrant influences were prevalent and ubiquitous. Populations in the upper watershed above partial natural barriers were highly distinct (*F*_ST_ = 0.093) and minimally impacted by apparent introgression. Genetic structure between watersheds paralleled differences in local demographics (e.g., variation in size), migratory restrictions, and habitat discontinuity. The evidence of restricted gene flow between putative remnant resident populations in the upper watershed and the admixed anadromous population in the lower watershed has implications for local steelhead productivity and regional conservation.

## 1 INTRODUCTION

The highly diverse life history of *Oncorhynchus mykiss* has been widely documented among populations throughout the species range along the North Pacific Rim (Beacham, Pollard, & Le, 1999; Busby et al., 1996; McPhee et al., 2007). Genetic differentiation varies broadly, with distinctions dictated by myriad factors including complex mating behaviors, habitat distribution, and the prevalence of plastic traits such as migration time (Crozier, Scheuerell, & Zabel, 2011; Hendry, Wenburg, Myers, & Hendry, 2002). Population distinctions are often more discernable at the regional level, in association with greater distances between spawning aggregates (Blankenship et al., 2011; Garza et al., 2014; Nielsen, Byrne, Graziano, & Kozfkay, 2009), but diverse life histories can lead to fine‐scale population distinctions (Benjamin, Connolly, Romine, & Perry, 2013; Thorpe, 2007), even within a discrete stream network (Kozfkay, Meyer, Schill, & Campbell, 2011; Nielsen, Pavey, Wiacek, & Williams, 2005). The migratory behavior of *O. mykiss* is one of the most influential life history attributes in terms of contributions to population genetic structure. Resident *O. mykiss* remain in fresh water from rearing to maturity, while conspecific anadromous steelhead trout migrate to the ocean where they typically grow for one to 3 years before returning to spawn in natal streams (Behnke, 2002). During rearing and spawning periods, the two forms typically co‐occur (sympatry) and compete for resources (Berejikian, Campbell, & Moore, 2013; Tartara & Berejikian, 2012) . Although resident spawners may give rise to anadromous offspring or vice versa, the prevalence of gene flow (i.e., degree of isolation) among the two sympatric population components may vary (Courter et al., 2013; Heath, Bettles, Jamieson, Stasiak, & Docker, 2008; Olsen, Wuttig, Fleming, Kretschmer, & Wenburg, 2006; Thrower, Hard, & Joyce, 2004; Zimmerman & Reeves, 2002).

Opportunities for reproductive interaction within and among *O. mykiss* migratory forms can be limited by a host of demographic influences, including relative abundances (Hilderbrand, 2003), size and age at maturity (e.g., complexity of mating systems; Seamons, Bentzen, & Quinn, 2004), and rate of repeat spawning or iteroparity (Keefer, Wertheimer, Evans, Boggs, & Peery, 2008). Concurrent temporal differences in run time and/or spawn time may lead to assortative mating (Hendry et al., 2002; Matala, French, Olsen, & Ardren, 2009; McMillan, Katz, & Pess, 2007). The distinctiveness of local populations can also be dramatically influenced by the landscape or fragmentation of habitat, particularly where dams and cataracts disrupt stream connectivity and limit migratory potential (Hayes et al., 2012; Narum, Zendt, Graves, & Sharp, 2008; Pearse et al., 2009). The intersection between watershed terrain, stream complexity, and flow regimes may produce a patchwork of rearing and spawning habitat that affects survival (Boughton, Fish, Pope, & Holt, 2009; Letcher, Nislow, Coombs, O'Donnell, & Dubreuil, 2007; Neville, Dunham, Rosenberger, Umek, & Nelson, 2009), propensity to migrate (Hecht, Campbell, Holecek, & Narum, 2013; Pavlov et al., 2008), and the degree of reproductive isolation among resident and anadromous *O. mykiss* phenotypes (Wellband, Atagi, Koehler, & Heath, 2012).

Introgression (i.e., reproductive interaction) may confound the genetic distinctiveness within or between natural spawning populations (Chilcote, Goodson, & Falcy, 2011; Small, Currens, Johnson, Frye, & Von Bargen, 2009), particularly in regions where hatchery stocking programs are prevalent. The influences and risks associated with introgression are likely to be relatively minor where hatchery‐origin and natural‐origin counterparts share similar life history and demographic characteristics (Gow, Tamkee, Heggenes, Wilson, & Taylor, 2011; Heggenes, Beere, Tamkee, & Taylor, 2006). The Pacific Northwest of the United States has a substantive history of *O. mykiss* hatchery stocking (Busby et al., 1996; Naish et al., 2008), including numerous subbasins in the Columbia River Basin. Tributaries that have remained exempt from direct hatchery intervention are nevertheless subject to potential indirect influences through immigration (i.e., straying of fish from exogenous sources; Keefer & Caudill, 2012; Westley, Quinn, & Dittman, 2013).

The Rock Creek Subbasin of Washington State, USA, is part of the middle Columbia River distinct population segment (DPS) where steelhead trout are currently listed as “threatened” under the Endangered Species Act (Busby et al., 1996; NMFS 2009 appendix C). Rock Creek and its tributaries are devoid of dams and other anthropogenic structural barriers, and opportunities to monitor the local *O. mykiss* population (e.g., placement of adult weir or juvenile traps) have been limited by the generally inaccessible landscape dominated by rangeland. Based on its geographic isolation, the Rock Creek subbasin is believed to support a distinct native population of steelhead trout (ICTRT 2003), but habitat conditions have had adverse effects on the abundance and productivity of the local fish population/s (WDF and WDW, 1993; Harvey 2014). Introgressive influences from nonlocal hatchery (or wild) fish remain generally unknown, and assumptions about the distinctiveness of *O. mykiss* in the Rock Creek subbasin have not been sufficiently verified through genetic monitoring or abundance surveys. In our study, we have conducted a genetic evaluation to characterize the Rock Creek steelhead trout population. We focused on testing three primary hypotheses: 1) the Rock Creek population is a single distinct population within the Columbia River Basin, 2) seasonally fragmented habitat does not influence genetic variation, and 3) potential natural barriers restrict gene flow between upper and lower sections of the watershed influencing sympatry between putative resident and anadromous life histories, respectively. Our objectives serve to inform regional conservation of the species. Understanding the degree of genetic distinctiveness in the Rock Creek population, where hatchery impacts are presumed small, will provide a more comprehensive understanding of immigrant or exogenous influences throughout the Columbia River Basin.

## 2 METHODS

### 2.1 Study area and steelhead habitat

**Figure 1:**
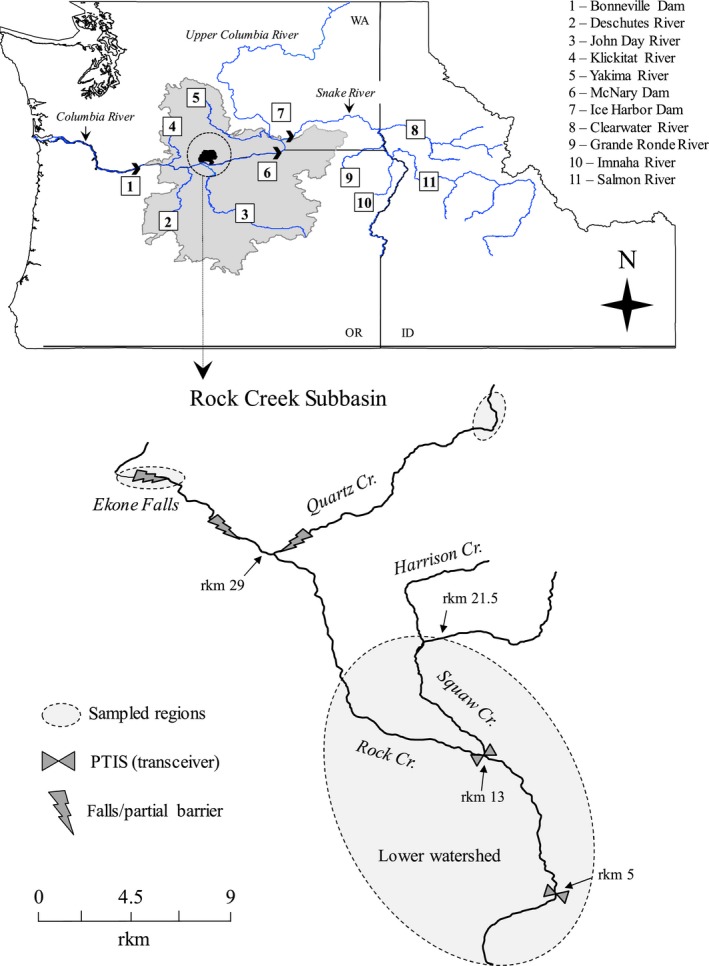
Map of the Rock Creek Subbasin in the Middle Columbia River DPS (shaded gray), located at latitude N45.704, longitude W120.464. Map identifiers include relevant locations and landmarks in the Columbia River Basin and major tributaries of the Snake River corresponding with reference reporting groups

Rock Creek is a tributary of the Columbia River located approximately 363 river kilometers (rkm) upstream of the Pacific Ocean. The small subbasin (~558 square kilometers) is dominated by rangeland, where developed land accounts for <1% of total area. The two main water courses within the subbasin are Rock Creek and Squaw Creek (Figure 1). There is no regulated flow and only minor water diversions to accommodate livestock watering and irrigation (Aspect Consulting and Watershed Professionals Network 2004; Harvey, 2014). The steep bedrock terrain contributes to short but intense flow responses following precipitation. Throughout the summer dry season, low flows and high water temperatures create long stretches of dewatered streambed, leaving a patchwork of isolated perennial pools. Unlike many watersheds in the Middle Columbia River region, Rock Creek has incurred no direct stocking of hatchery fish.

The migratory limit of anadromous steelhead occurs approximately 0.5 rkm above the Quartz Creek confluence with Rock Creek (rkm 28.6), and approximately 1.3rkm upstream of the Squaw Creek/Harrison Creek confluence (rkm 20.3; Figure 1). The historical range of anadromy in Rock Creek is largely unknown. During spawning surveys between 2009 and 2014, we observed the majority of steelhead redds in the mainstem of Rock Creek below rkm 22, and in Squaw Creek 9 rkm upstream of the Rock Creek confluence (Harvey, 2014). Habitat surveys were conducted in September 2011 and September to October 2012 throughout most of the anadromous fish‐bearing reaches of Rock Creek to determine the spatial distribution and seasonal use of habitat by juvenile O. *mykiss* prior to smolt outmigration in spring (Allen, Munz, & Harvey, 2014; Harvey, 2014). Sections of stream were partitioned into three stream categories: 1) nonpool dry, defined as dewatered areas, 2) nonpool wet—defined as wetted gravel but no pools, and 3) perennial pool habitat. The latter represents rearing and refuge habitat during the dry season. Latitude and longitude coordinates were recorded at transitions (way‐points) between habitat categories, and habitat was mapped to the stream network using ArcMap version 10.0 (copyright © 1999‐2010 ESRI Inc.).

### 2.2 Sampling and data collection

**Figure 2:**
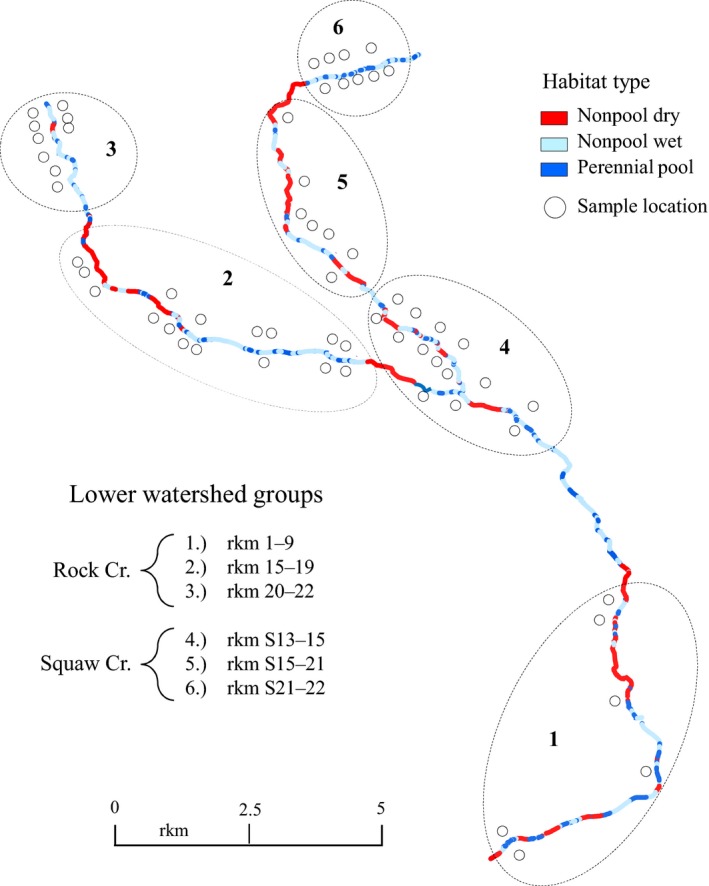
Map showing the distribution of designated habitat types mapped by waypoint in 2011 and 2012. Sample locations along the stream (2008–2012) are grouped within six stream sections that are loosely bounded by stretches of dry river bed and breaks in coverage in the lower watershed of the Rock Creek Subbasin

The absence of weirs or traps in Rock Creek precluded sampling of adult steelhead, except for four postspawn steelhead mortalities sampled during redd surveys conducted in 2009 and 2010. Electrofishing was used to sample juvenile *O. mykiss* from pool habitat in the lower watershed each spring and early fall between 2008 and 2012 in coordination with PIT‐tag population assessment surveys. Juvenile age categories were inferred from fork length (FL) size measurements (Table 1). Fish <70 mm in spring and <90 mm in the fall were deemed age‐0, larger fish up to 149 mm were grouped as age‐1+ (see Gallagher, 2004 for comparison), and those at least 150 mm FL were presumed to be mature age2+ *O. mykiss*. Fish ≥70 mm were PIT‐tagged following accepted procedures (Columbia Basin Fish and Wildlife Authority 1999), and juvenile *O. mykiss* and adult steelhead movement was monitored between 2009 and 2012 using PTIS transceivers installed at rkm 5 and rkm 13 of Rock Creek (Figure 1). Large numbers of juveniles were handled and interrogated for PIT tags (*n *=* *6,218). Sampling for genetic analyses was limited by encounter rate and permitting (as per NOAA fisheries). We targeted a random sample of five age‐0 and five age‐1+ juveniles from each pool, and all age‐2+ fish, which were less abundant. Samples were grouped by spatial distribution into stream sections loosely bounded by extended stretches of “nonpool dry” habitat (i.e., dewatered river bed) or gaps in sampling coverage (Figure 1). The resulting six lower watershed groups were identified by upstream distance from the Columbia River confluence: rkm1‐9, rkm15‐19, and rkm20‐22 in Rock Creek and rkmS13‐15, rkmS15‐21, and rkmS21‐22 in Squaw Creek (Figure 2). Sample sizes among sections ranged from *n *=* *47 to *n *=* *154 (Table 1). Two locations were sampled in the upper watershed of Rock Creek, beyond the presumed limit of anadromy where resident *O. mykiss* may reside (Courter, Justice, & Cramer, 2009). One collection included a combination of fish sampled directly above and below Ekone falls located at ~rkm 33 of Rock Creek (*n *=* *56; Figure 1). A second collection was sampled approximately 12 rkm upstream of a partial barrier in Quartz Creek (*n *=* *21).

**Table 1:** Summary statistics for the Rock Creek *Oncorhynchus mykiss* genetic sample. Samples are grouped by sample year and river section within watershed. Age proportions were inferred from fork lengths: age‐1+ ≥70 mm in spring and ≥90 mm in the fall. Smaller fish were designated age‐0. Fish ≥150 mm FL were deemed age‐2+ or “mature.” Mean genetic diversity is observed (*H*_O_) and expected (*H*_E_) heterozygosity. Differences in age structure within and between watersheds are shaded gray

Statistic	Lower watershed (Rock Cr.)			Lower watershed (Squaw Cr.)				Upper watershed		
	rkm1‐9	rkm15‐19	rkm 20‐22	rkmS13‐15	rkmS15‐21	rkmS21‐22	Overall	Ekone	Quartz	Overall
Genetic sample size (*n*)										
2008	33	0	51	70	0	0	154	0	0	0
2009	0	20	11	0	19	8	58	0	0	0
2010	0	12	0	20	11	11	54	0	0	0
2011	21	49	35	42	11	28	186	25	0	25
2012	11	12	5	22	48	0	98	0	0	0
2013	0	0	0	0	0	0	0	31	21	52
**Total**	**65**	**93**	**102**	**154**	**89**	**47**	**550**	**56**	**21**	**77**
Male (%)	0.42	0.47	0.60	0.44	0.59	0.39	0.49	0.53	0.57	0.55
Age proportions										
age‐0	0.72	0.73	0.24	0.65	0.62	0.35	0.59	0.12	0.00	0.10
age‐1+	0.28	0.27	0.76	0.35	0.38	0.65	0.41	0.88	1.00	0.90
mature	0.11	0.08	0.10	0.09	0.09	0.05	0.08	0.32	0.48	0.35
Relatedness (%)										
Unrelated	0.95	0.95	0.94	0.94	0.95	0.94	0.94	0.89	0.90	0.90
Half‐sibling	0.04	0.05	0.05	0.05	0.05	0.05	0.05	0.08	0.04	0.06
Full‐sibling	0.01	0.00	0.01	0.01	0.00	0.01	0.01	0.02	0.04	0.03
Parent/offspring	0.00	0.00	0.00	0.00	0.00	0.00	0.00	0.01	0.02	0.02
Heterozygosity										
*H*_O_	0.31	0.31	0.31	0.31	0.32	0.32	0.31	0.29	0.28	0.29
*H*_E_	0.31	0.31	0.32	0.31	0.31	0.32	0.31	0.28	0.29	0.28

### 2.3 Laboratory protocol and genetic analyses

Genomic DNA was extracted using a standard Qiagen^®^ DNeasy™ protocol. Samples were genotyped at 192 SNP loci using TaqMan chemistry (Life Technologies) and Fluidigm 96.96 dynamic array chips. The SNP panels were developed by multiple sources (Ackerman & Campbell, 2012; Matala, Ackerman, Campbell, & Narum, 2014). Polymerase chain reaction (PCR) amplification followed the protocol of Hess, Campbell, Matala, and Narum (2012). Quality control measures allowed for no more than 10% missing data per individual. The panel included three species diagnostic markers used for hybrid screening between *O. clarkii* (cutthroat trout) and *O. mykiss*, and a sex‐determining SNP locus (*OmyY1_2SEXY*) provided identification of genetic sex.

Allele frequency and heterozygosity were estimated using GenAlEx version 6.5 (Peakall & Smouse, 2006). Deviations from Hardy–Weinberg equilibrium (HWE) expectations, and tests of linkage disequilibrium were evaluated using GENEPOP v. 3.3 (Raymond & Rousset, 1995), applying an adjusted significance level for multiple tests (Benjamini & Yekutieli, 2001; Narum, 2006). The program ARLEQUIN version 3.5 (Excoffier, Laval, & Schneider, 2005) was used to calculate pairwise population *F*_ST_ among sample years and among river sections to evaluate temporal and spatial differentiation, respectively. A pairwise matrix of Nei's standard genetic distance (Nei, 1972) and an unrooted neighbor‐joining (NJ) tree were generated using PHYLIP version 3.68 (Felsenstein, 2008). Relatedness was estimated using the program ML‐Relate (Kalinowski, Wagner, & Taper, 2006) to test for potential kinship bias in the form of full‐sibling groups within each stream section.

The *O. mykiss* population in Rock Creek belongs to the inland genetic lineage (*O. m. gairdneri*) commonly referred to as redband trout. Stocking of hatchery rainbow trout (*O. m. irrideus*) belonging to the distinct coastal genetic lineage has occurred in some locations within the inland region of the Columbia River Basin. We evaluated genetic structure between Rock Creek *O. mykiss* and 24 rainbow trout reference populations utilizing complimentary genotypic data from Matala et al. (2014) to test for evidence of prior hatchery stocking. Pairwise genetic distances generated in GenAlEx version 6.5 were used to display population clustering patterns in a principal coordinates analysis (PCoA) plot.

We evaluated the distinctiveness of Rock Creek *O. mykiss* among populations of the Columbia River Basin using genetic stock identification (GSI). Reference populations for assignment tests included 53 inland lineage steelhead populations (Matala et al., 2014), combined into seven previously defined reporting groups (Hasselman et al., 2016; Hess, Ackerman, et al., 2016), including the Upper Columbia River and five discrete Snake River groups: South Fork Salmon River (SFSALM), Middle fork Salmon River (MFSALM), upper Salmon River (UPSALM), South Fork Clearwater River (SFCLWR), and upper Clearwater River (UPCLWR; Table S1). The seventh reporting group (MGILCS) encompasses genetically similar populations spanning the middle Columbia River to tributaries of the lower Snake River, including the Imnaha River, Grande Ronde River, and lower Clearwater River (Hess et al. 2015; Figure 1). Although Rock Creek is located within the MGILCS geographic region, there is no information confirming the local population is genetically similar to MGILCS populations. Therefore, the upper and lower watershed groups from Rock Creek were each treated as a unique reporting group. Individual assignment likelihood scores (LS) were generated using GENECLASS2 (Piry et al., 2004), implementing the Bayesian method of Rannala and Mountain (1997) in a leave‐one‐out jackknife procedure. The assigned origin of each individual was the population with highest corresponding GSI likelihood score from among the top five ranked scores. The rate of self‐assignment was defined as the proportion of fish sampled in Rock Creek that genetically assigned to Rock Creek (specific to watershed), and results were partitioned between the upper and lower watershed samples to infer the degree of gene flow between the two. Assignments to out‐of‐basin reference populations were used to infer immigrant (i.e., stray) influences in the Rock Creek population. Lastly, we used GSI to identify the origins of four steelhead carcasses, and 11 upstream migrating adult steelhead that were PIT‐tagged at Bonneville Dam (see Figure 1) prior to being detected at PTIS transceivers in Rock Creek. Genotypes for Bonneville fish were available from the Columbia River Inter‐Tribal Fish Commission (pers. comm. Jon Hess).

## 3 RESULTS

### 3.1 Habitat and demographic descriptions

The distribution of dewatered stream sections and dispersed perennial pools was consistent over two survey seasons, suggesting interannual variation in stream hydration patterns may be minimal from early summer to early fall (Figure 2). The average proportion of age1+ *O. mykiss* among lower watershed groups ranged from 6% (rmk1‐9) to 86% (rkm20‐22) in the spring and from 18% (rkm15‐19) to 66% (rkm20‐22) in the fall (Table S2). The average age‐1+ proportions in the upper watershed groups were 59% and 53% in the spring and fall, respectively. The proportion of fish among lower watershed groups that were considered reproductively mature ranged from a low of 2% to 6% in the spring, and from 6% to 13% in the fall. In the upper watershed, the proportion of mature fish in the sample ranged from 31% in the spring (Ekone Falls) to 48% in the fall (Quartz Creek). Relative proportions of age‐0 and age‐1+ *O. mykiss* in the genetic subsample (Table 1) had a similar age structure as the survey sample, with a similar trend toward larger fish observed farther upstream. The proportion of male *O. mykiss* in the lower watershed ranged from 39% (rkmS21‐22) to 60% (rkm20‐22). In the upper watershed, the male proportion was 53% at Ekone Falls and 57% in Quartz Cr. (Table 1b).

A total of 3,088 juvenile *O. mykiss* were PIT‐tagged during electrofishing surveys, and 27% (832) were subsequently detected at downstream PTIS transceivers in Rock Creek or the Columbia River. Juveniles tagged downstream of rkm 13 were observed outmigrating at a rate of 39% to 46%. Fish tagged upstream of rkm 13 were detected at a significantly lower rate (26%–28%; *p *<* *.0001); however, there was no observed difference in rate of outmigration between Squaw Creek (26%) and Rock Creek (28%). Conversely, none of the *O. mykiss* tagged in the upper watershed at Ekone Falls (*n *=* *69) and Quartz Creek (*n *=* *21) were detected at a downstream PTIS.

Prior to the spawning seasons from 2010 to 2013, there were 37 different adult steelhead detected at PTIS's in Rock Creek, including 11 steelhead tagged at Bonneville Dam. GSI results revealed that two of 11 originated from Rock Creek (LS = 93.6, LS = 97.1), while the other nine were assigned as out‐of‐basin strays (Table S3). The remaining 26 adult tag detections included seven fish that had been PIT‐tagged in Rock Creek as juveniles, and 19 with known mark/release locations in the Snake River basin (PSMFC, 2009; Table S3). Several of the tagged individuals were initially detected migrating upstream of Rock Creek in the Columbia River as far as Ice Harbor Dam in the Snake River (~268 rkm beyond Rock Creek; Figure 1) before returning back downstream to Rock Creek. Among four recovered adult steelhead carcasses, one was assigned to the SFCLWR reporting group (LS = 96.0), and three were assigned to Rock Creek (LS = 95.0, LS = 96.0, and LS = 51.4).

### 3.2 Genetic population structure and GSI

**Figure 3:**
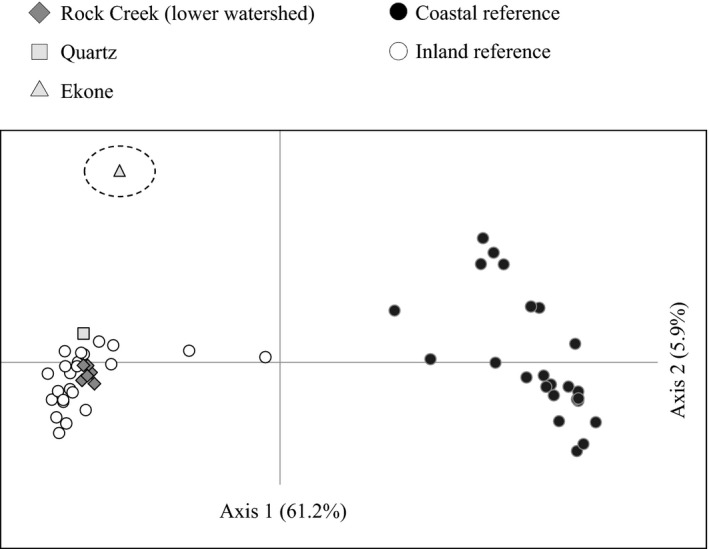
Principal coordinates analysis (PCoA) plot. The ellipse highlights the distinct Ekone Falls collection among distinct clusters of inland lineage and coastal lineage populations from throughout the Columbia River Basin; data available from Matala et al. (2014)

Following exclusion of loci for linkage disequilibrium and HWE deviations, 180 SNPs were retained for population genetic analyses. Allelic diversity was similar between upper and lower watershed groups (mean *H*_O_ = 0.28, and *H*_O_ = 0.31, respectively). Species diagnostic markers confirmed all sampled individuals were *O. mykiss,* and there was no evidence of hybridization with cutthroat trout. Genetic distance clustering in the PCoA plot definitively confirmed inland lineage origin for all collections from the Rock Creek subbasin, which clustered tightly with inland lineage reference populations, and distinctly from coastal lineage reference populations (Figure 3). Kinship analyses revealed that the proportion of unrelated individuals ranged from 89% in the Ekone Falls group to 95% in three different lower watershed groups (Table 1). Full‐sibling proportions ranged from 8% at Ekone Falls to 4%–5% among all remaining groups. Results were variable among sites but nominally different, with no apparent upstream kinship bias.

Temporally based genetic variation between lower watershed collections spanning 2008–2012 ranged from *F*_ST_ = 0.000 to *F*_ST_ = 0.005, with significant variation occurring primarily in comparisons involving the 2008 sample year (Table 2a). All temporal comparisons between upper and lower watersheds were significant regardless of sample year (pairwise *F*_ST_ values ranging from 0.031 to 0.070). Spatially based genetic variation between six lower watershed collections was relatively small (*F*_ST_ range 0.001 to 0.008), but six of nine comparisons between Squaw Creek and Rock Creek stream sections indicated significant differentiation (Table 2b). All spatial comparisons between upper and lower watersheds were significant and consistently an order of magnitude larger (mean *F*_ST_ = 0.051; Table 2b) than comparisons among lower watershed groups. The greatest genetic differentiation was observed between the two upper watershed groups from Ekone Falls and Quartz Creek (*F*_ST_ = 0.093).

**Table 2:** Summary of among‐group genetic variation (*F*_ST_). Pairwise comparisons were evaluated for (a) samples grouped temporally (by year) across all lower watershed groups, and (b) samples grouped spatially by lower watershed river section. *F*_ST_ values are in the lower half‐matrix with corresponding *p*‐values are in the upper half‐matrix. Significant values are bolded (*p *<* *.001)

(a) Temporal	Lower watershed					Upper watershed	
	2008	2009	2010	2011	2012	Ekone	Quartz
2008	–	0.277	0.000	0.000	0.000	0.000	0.000
2009	0.000	–	0.024	0.000	0.039	0.000	0.000
2010	**0.003**	0.002	–	0.021	0.001	0.000	0.000
2011	**0.005**	**0.004**	0.002	–	0.342	0.000	0.000
2012	**0.003**	0.002	0.003	0.000	–	0.000	0.000
Ekone	**0.065**	**0.065**	**0.070**	**0.067**	**0.062**	–	0.000
Quartz	**0.031**	**0.034**	**0.032**	**0.040**	**0.036**	**0.093**	–

(b) Spatial	Lower watershed (Rock Cr.)			Lower watershed (Squaw Cr.)			Upper watershed	
	rkm1‐9	rkm15‐19	rkm20‐22	rkmS13‐15	rkmS15‐21	rkmS21‐22	Ekone	Quartz
rkm1‐9	–	0.055	0.046	0.010	0.000	0.000	0.000	0.000
rkm15‐19	0.001	–	0.003	0.004	0.003	0.000	0.000	0.000
rkm20‐22	0.002	0.002	–	0.000	0.000	0.000	0.000	0.000
rkmS13‐15	0.002	0.002	**0.004**	–	0.000	0.000	0.000	0.000
rkmS15‐21	**0.004**	0.002	**0.003**	**0.002**	–	0.010	0.000	0.000
rkmS21‐22	**0.008**	**0.005**	**0.007**	**0.006**	0.003	–	0.028	0.000
Ekone	**0.067**	**0.063**	**0.064**	**0.069**	**0.063**	0.071	–	0.000
Quartz	**0.042**	**0.037**	**0.032**	**0.036**	**0.035**	**0.038**	**0.093**	–

**Figure 4:**
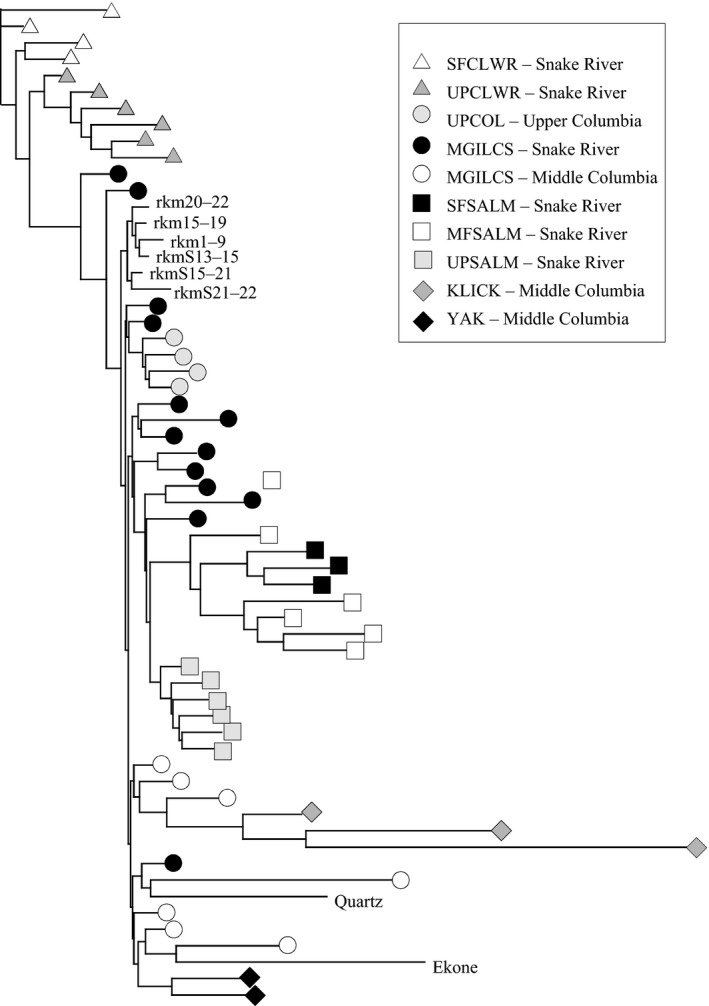
Neighbor‐joining tree based on Nei's genetic distance. Rock Creek Subbasin groups are identified by river reach or upper watershed location. Reference populations were defined by reporting groups as follows: Klickitat River (KLICK), Yakima River (YAK), a composite group of Middle Columbia River populations and Lower Snake River populations (MGILCS), the Upper Columbia River (UPCOL), Upper Clearwater River (UPCLWR), South Fork Clearwater (SFCLWR), middle and south forks of the Salmon River (MFSALM and SFSALM), and Upper Salmon River (UPSALM). For additional details regarding reference populations see Matala et al. (2014) and Hess, Ackerman, et al., 2016

Spatial clustering in the NJ tree topology indicated significant genetic distance between the upper and lower watershed groups in Rock Creek (Figure 4). Ekone Falls and Quartz Creek groups were most similar to reference populations from the Middle Columbia River region, while lower watershed groups appeared most similar to several regions in the Snake River Basin.

Results of GSI analyses revealed a self‐assignment rate of 75% (mean LS = 92.3) for the Ekone Falls group, and 67% (mean LS = 96.0) for the Quartz Creek group. The next most frequently assigned origin was to the MGILCS reporting group, and only three individuals from Ekone and Quartz groups assigned to the lower watershed of Rock Creek with highest likelihood. (Table 3). Assigned origins among six lower watershed groups were dominated by self‐assignments, ranging from 54% to 61%, however; corresponding likelihood scores were low ranging from LS = 69.6 to LS = 81.6. The largest proportion of out‐of‐basin assignments among all Rock Creek lower watershed groups were to the MGILCS and UPSALM reporting groups. Only four individuals sampled in the lower watershed (0.7%) were assigned to either the Ekone Falls or Quartz Creek groups in the upper watershed, including three in the farthest upstream group (rkm20‐22) and one in the farthest downstream (rkm1‐9; Table 3).

**Table 3:** Results of GSI assignment tests. Assigned origins of Rock Creek samples are identified by the proportion assigned (%) to each out‐of‐basin reporting group with corresponding mean likelihood score (LS). The largest out‐of‐basin proportions are shaded gray; some results are based on single observations (*). Self‐assignment rates are bolded

Group		Out‐of‐basin reference									Self‐assignment		
		UPCLWR	SFCLWR	MFSALM	SFSALM	UPSALM	MGILCS	UPCOL	KLICK	YAK	ROCK	Ekone	Quartz
Lower watershed													
rkm1‐9	%	0.02*	0.09	0	0	0.12	0.11	0.05	0	0	**0.60**	0.02*	0
	LS	66.5	81.1	–	–	71.9	60.2	60.8	–	–	**80.9**	41.2	–
rkm15‐19	%	0	0.03	0	0.01*	0.17	0.18	0.03	0	0.02	**0.55**	0	0
	LS	–	74.3	–	60.5	68.6	66.8	49.5	–	74.5	**79.7**	–	–
rkm20‐22	%	0.01*	0.03	0	0.02	0.10	0.22	0.02	0	0.04	**0.54**	0.01*	0.02
	LS	99.4	60.1	–	76.4	60.9	62.4	69.6	–	73.6	**73.5**	83.2	60.7
rkmS13‐15	%	0.02	0.03	0.02	0	0.10	0.15	0.06	0	0.01	**0.60**	0	0
	LS	55.5	94.6	64.7	–	68.6	61.6	62.5	–	46.6	**81.6**	–	–
rkmS15‐21	%	0.01*	0.02	0	0.01*	0.09	0.17	0.03	0	0.06	**0.61**	0	0
	LS	30.1	95.6	–	76.8	64.4	54.7	65.2	–	60.9	**74.3**	–	–
rkmS21‐22	%	0	0.02*	0	0.02*	0.13	0.13	0.11	0	0.04	**0.55**	0	0
	LS	–	79.9	–	49.1	74.7	59.3	61.0	–	57.5	**69.6**	–	–
Upper watershed													
Ekone	%	0	0	0	0	0	0.14	0.02*	0.04	0.02*	0.04	**0.75**	**0**
	LS	–	–	–	–	–	75.2	60.8	71.7	95.8	45.1	**92.3**	–
Quartz	%	0	0	0	0	0	0.19	0.05*	0	0.05*	0.05*	**0**	**0.67**
	LS	–	–	–	–	–	61.7	56.1	–	47.5	58.5	–	**96.0**

## 4 DISCUSSION

### 4.1 Gene flow and immigration in the lower watershed

The genetic structure among *O. mykiss* in the lower watershed of Rock Creek was spatially consistent, with limited differentiation among groups. In our stock identification analyses, we saw variable results with regard to out‐of‐basin influences in Rock Creek. Some steelhead detected at PIT‐tag transceivers in Rock Creek had been previously tagged as juveniles in Rock Creek, suggesting that steelhead continue to spawn naturally in Rock Creek. Further studies would be necessary to gauge the abundance or effective population size of a putative native spawning population, but it is likely to be small given the availability of spawning habitat. However, the moderate likelihood scores for self‐assignments as well as significant numbers of out‐of‐basin assignments point to a clear lack of genetic distinction of the Rock Creek population within the Columbia River region. The insufficient power to confidently (i.e., LS > 0.9) assign origins of fish sampled from Rock Creek is consistent with results for adjacent Middle Columbia River populations. Minimal GSI resolution has been influenced by significant regional straying or admixture (see Hess, Hess, 2016), likely contributing to the broad geographic expanse of the MGILCS reporting group (Hess, Campbell, et al., 2015; Hess, Ackerman, et al., 2016). In fact, most PIT‐tag detections in Rock Creek were from individuals that had known out‐of‐basin juvenile release locations, and the probable reproductive success of immigrants is manifest as significant admixture in the lower watershed (Araujo, Candy, Beacham, White, & Wallace, 2014; Hess & Matala, 2014). No sampling was conducted beyond rkm 22 in the lower watershed, but population panmixia likely extends upstream to rkm 29 at the site of a partial barrier falls as steelhead migration through the lower stream corridor is not constrained by habitat. Differences in immigrant origins among stream sections in the lower watershed may account for minor genetic variation observed between the upper reaches of both Squaw Creek and Rock Creek. In contrast, the distinctiveness of upper watershed groups (upstream of rkm 28.6) indicates an effective buffering from introgression relative to upstream distance. Ekone Falls and Quartz Creek samples had highly confident self‐assignments based on likelihood scores, and included no assignments to the lower watershed of Rock Creek, with relatively few out‐of‐basin assignments (having low likelihood).

Over the past 25–50 years, hatchery stocking has been used extensively in the Columbia River Basin for fishery enhancement and harvest opportunities, or for supplementation to aid steelhead conservation and recovery goals (Paquet et al., 2011). However, research has shown that hatchery straying from both types of programs can influence local population structure in salmonids (e.g., Hess & Matala, 2014; Van Doornik, Berejikian, & Campbell, 2013; Van Doornik, Eddy, et al., 2013). Significant straying of nonlocal hatchery stocks has been observed in large subbasins adjacent to Rock Creek including the Deschutes River (Hand & Olson, 2003; Hess, Hess, et al., 2016) with its own local hatchery program, and the John Day River (Ruzycki & Carmichael, 2010; Figure 1) with no direct hatchery stocking. Hatchery steelhead are believed to exhibit a higher stray rate compared to natural‐origin steelhead (Keefer & Caudill, 2014; Quinn, 1993), but immigrants in Rock Creek identified via PIT‐tag detection revealed that most (26 of 37) were natural‐origin steelhead (Table S3). One caveat to consider is that not all hatcheries physically mark (e.g., adipose fin clip) fish, which is used to distinguish them from wild fish. The absence of population trend data or archival samples in our study precluded us from accurately assessing historical steelhead immigration rates in Rock Creek or the relative impacts associates with supplementation activity. Nevertheless, the accumulated effects of introgression over an unknown number of generations have likely diminished our ability to confidently differentiate local from exogenous sources of anadromous productivity in the lower watershed of Rock Creek.

### 4.2 Migratory life history differences among watersheds

Overall, the evidence from our study suggests that *O. mykiss* in the upper watershed of Rock Creek are native resident populations that have undergone significant genetic drift in isolation from an admixed anadromous population occupying the lower watershed. Significant heterogeneity was observed between the lower watershed groups and the populations above putative barriers at Ekone Falls and Quartz Creek. Typically, the resident *O. mykiss* phenotype contributes to the overall productivity of sympatric anadromous steelhead. Resident trout may produce progeny that undergo smoltification and migrate to sea, while offspring of anadromous steelhead can adopt a nonmigrator, resident life history (Courter et al., 2013). However, gene flow between resident and anadromous population components is contingent to a large degree on their respective distributions (Narum et al., 2008; Sogard et al., 2012). Partial barriers, variable flows and other physical elements of stream habitat can restrict movement and influence opportunities for interactions. Based on characteristics of both Ekone Falls and the falls in Quartz Creek (e.g., height, flow, plunge pools), it is thought that neither is a complete barrier to upstream migrating steelhead, but we could not conclusively confirm this. Even with obstructed upstream movement, individuals in upstream reaches can become entrained (fall) or swim downstream (Hayes et al., 2012; Pearse et al., 2009; Van Doornik, Berejikian, et al., 2013; Van Doornik, Eddy, et al., 2013). In fact, approximately half of our Ekone Falls samples were taken below the falls. It would therefore be reasonable to assume that unidirectional gene flow in a downstream direction could occur. However, limited gene flow was evident between watersheds in our analyses.

Growth trajectory, generation time, and body size can affect the propensity to migrate (Benjamin et al., 2013; Letcher et al., 2007; Sogard et al., 2012), and upper watershed groups were dominated by larger, putatively mature fish. From an evolutionary perspective, it is likely that selection operates against migratory phenotypes in above barrier populations (Pearse et al., 2009) because fish that descend barriers become isolated from natal upstream reaches, resulting in lost productivity in the source population. Despite nonmigratory behaviors, it may be possible for populations above barriers to maintain dormant migratory traits for long periods of time (Holecek & Scarnecchia, 2013; Thrower et al., 2004). Sex ratios have proven useful for predicting parental life history (anadromous vs. resident) among outmigrating *O. mykiss* juveniles (Ohms, Sloat, Reeves, Jordan, & Dunham, 2014). Rundio et al. (2012) found that sex ratios of outmigrants were skewed toward females in a sympatric resident and anadromous population. However, Van Doornik, Berejikian, et al., (2013), Van Doornik, Eddy, et al., (2013) showed that male resident *O. mykiss* have a greater tendency to descend falls, inferring that sex ratios are likely to be skewed in favor of females in above barrier populations where sympatry between resident and anadromous fish is limited. Gender ratios in the upper watershed of Rock Creek were only slightly greater than 50% male and comparable to the lower watershed groups, thus limiting our ability to infer relative life history origins based on sex ratios (see Ohms et al., 2014).

There was no confirmed emigration of *O. mykiss* from the upper watershed of Rock Creek based on PIT‐tag detections, but four individuals in the lower watershed were assigned to the upper watershed. It may be that fish in the upper watershed do indeed move downstream to some degree, but that they subsequently suffer a high rate of mortality. The seasonally dry river bed, low flows and warmer water temperatures typical of the lower watershed represent significant factors known to have dramatic effect on habitat use strategies or oversummer survival of juvenile salmonids (Grantham, Newburn, McCarthy, & Merenlender, 2012; May & Lee, 2004; Mills et al., 2012; Walsworth, Schindler, Griffiths, & Zimmerman, 2014). Larger fish (i.e., age‐1+) in the lower watershed were concentrated in the farthest upstream sections (rkm 20‐22 and rkmS21‐22), where plentiful perennial pool habitat may facilitate higher oversummer survival. However, the stream progresses toward significantly cooler and wetter conditions and more stable flows in the upper watershed. These habitat distinctions have important demographic implications because environmental conditions and juvenile densities can affect migratory plasticity (Agrawal, 2001; Courter et al., 2013; Crozier et al., 2011; Mills et al., 2012; Stearns, 1989) but also the frequency of ecological interactions that induce slower growth and higher mortality (McMichael & Pearsons, 2001; McMichael, Sharpe, & Pearsons, 1997). Individuals moving downstream between watersheds might experience phenotypic incompatibility with local conditions (i.e., thermal tolerances; Narum, Campbell, Kozfkay, & Meyer, 2010; Narum, Campbell, Meyer, Miller, & Hardy, 2013) and regulated behavior in response to the habitat (Reeves, Grunbaum, & Lang, 2009) that leads to an elevated risk of mortality. Moreover, downstream migrating fish may be unaccustomed to optimal feeding behavior, prey densities, disease exposure and other habitat variables in the lower watershed (Pearsons, Phelps, Martin, Bartrand, & McMichael, 2007; Schroeder, 2007). Finally, assortative mating or asynchrony in spawning time (Donohoe, Adams, & Royer, 2008; Narum et al., 2008; Zimmerman & Reeves, 2000) may restrict gene flow between upper and lower watershed populations.

We have provided additional insight into *O. mykiss* population structure and life history variation that is applicable throughout the species range, but particularly within the Columbia River Basin. Our results contribute to a broader understanding of the rangewide prevalence of steelhead straying and dispersal. Immigrant influences observed within unstocked and minimally managed watersheds such as Rock Creek may serve as a prudent reference for gauging expected influences on natural spawning populations in more heavily managed subbasins. The complex issues surrounding life history diversity and sympatry among *O. mykiss* populations cannot be easily generalized and continue to challenge managers. Fisheries literature is replete with examples of sympatry and gene flow between resident and anadromous *O. mykiss* populations. For sympatric, interbreeding forms, the resident life history is typically viewed as a genetic “cache” to be considered in conservation and recovery planning for declining steelhead populations (Hayes et al., 2012; Holecek & Scarnecchia, 2013; McPhee et al., 2007; Van Doornik, Berejikian, et al., 2013; Van Doornik, Eddy, et al., 2013; Weigel, Connolly, & Powell, 2014). There are, however, local or watershed‐specific exceptions as exemplified in our study in which different migratory phenotypes experience restricted gene flow (Docker & Heath, 2003; McMillan et al., 2007; Narum, Contor, Talbot, & Powell, 2004). Conservation perspectives aimed at those populations should be cognizant of associated productivity distinctions and their contributions to overall productivity among large management units. Application of new research avenues involving adaptive variation and markers associated with anadromy (Hale et al. 2013; Hecht, Thrower, Hale, Miller, & Nichols, 2012; Limborg et al., 2012; Matala, Hess, & Narum, 2011; Narum et al., 2011; Nichols, Edo, Wheeler, & Thorgaard, 2008; Pearse, Miller, Abadia‐Cardoso, & Garza, 2014) would likely elucidate the role of inherent and external factors governing migratory behavior of *O. mykiss* in Rock Creek.

## AUTHORS CONTRIBUTION

Dr. Shawn Narum served as supervisory geneticist, providing conceptual and analytical research support in this study, and was a primary editor of the submitted draft manuscript. Brady Allen made substantial contributions to writing, and was the primary field biologist coordinating field surveys and overseeing collection of field data. Elaine Harvey was the primary study designer and planner. Ms. Harvey obtained permits, established contracts, was instrumental in data collection, and contributed to writing. Andrew Matala conducted all genetic analyses, coordinated field and laboratory components of the study, made contributions in data collection, and acted as primary author in the drafting of this manuscript.

## CONFLICT OF INTEREST

None declared.
