# Supplementary material for: Restricted gene flow between resident Oncorhynchus mykiss and an admixed population of anadromous steelhead
Source: Ecol Evol. 2017 Sep 8;7(20):8349–62. doi: 10.1002/ece3.3338 (PMC5648649; doi:10.1002/ece3.3338)
Supplement: Supplementary file 1 [file ECE3-7-8349-s001.docx]

**Restricted gene flow between resident *Oncorhynchus mykiss* and an admixed population of anadromous steelhead**

Andrew P. Matala^1*^, Brady Allen^2^, Shawn Narum^1^, and Elaine Harvey^3^

^1^Columbia River Inter-Tribal Fish Commission

Hagerman Fish Culture Experiment Station

3059-F National Fish Hatchery Road

Hagerman, ID 83332, USA

*corresponding author: [mata@critfc.org](mailto:mata@critfc.org)

^2^ USGS-Western Fisheries Research Center

Columbia River Research Laboratory
5501A Cook-Underwood Rd.
Cook, WA 98605

Current: Bonneville Power Administration

P.O. Box 3621
Portland, OR 97208-3621

^3^Yakama Nation Fisheries

4 Bickleton Hwy.

Goldendale,WA 98620

**Keywords:** resident redband trout, steelhead trout, sympatry, admixture, habitat heterogeneity, immigration, Columbia River,

**Running Title:** habitat discontinuity and immigration influence *O. mykiss* genetic structure

Peer Review Disclaimer: This draft manuscript is distributed solely for purposes of scientific peer review. Its content is deliberative and predecisional, so it must not be disclosed or released by reviewers. Because the manuscript has not yet been approved for publication by the U.S. Geological Survey (USGS), it does not represent any official USGS finding or policy.

Table S1. Reference populations from the Columbia River Basin used in GSI assignment tests. Population identification numbers are as reported in Matala et al. (2014).

| population ID | n | lineage | reporting group | region/subbasin | stream |
| --- | --- | --- | --- | --- | --- |
|  |  |  |  |  |  |
| POP01 | 50 | coastal | N/A | Quinault R. | Quinault R. |
| POP02 | 50 | coastal | N/A | Willamette R. | Clackamas R. |
| POP03 | 47 | coastal | N/A | Willamette R. | Eagle Cr. |
| POP04 | 43 | coastal | N/A | Willamette R. | N. F. Eagle Cr. |
| POP05 | 50 | coastal | N/A | Willamette R. | Skamania Stock |
| POP06 | 50 | coastal | N/A | Willamette R. | Little Rock/Mad Cr. |
| POP07 | 39 | coastal | N/A | Willamette R. | N. F. Santiam/ Mad Cr. |
| POP08 | 50 | coastal | N/A | Willamette R. | S. F. Santiam/ Wiley Cr. |
| POP09 | 25 | coastal | N/A | Willamette R. | Canyon Cr. |
| POP10 | 26 | coastal | N/A | Willamette R. | Luckiamute Cr. |
| POP11 | 30 | coastal | N/A | Willamette R. | Willamina Cr. |
| POP12 | 50 | coastal | N/A | Abernathy Cr. | Abernathy Cr. |
| POP13 | 45 | coastal | N/A | Coweeman R. | Coweeman R. |
| POP14 | 50 | coastal | N/A | Cowlitz R. | Cowlitz R. |
| POP15 | 47 | coastal | N/A | Germany R. | Germany Cr. |
| POP16 | 50 | coastal | N/A | Kalama R. | Kalama R. |
| POP17 | 50 | coastal | N/A | Kalama R. | Kalama R. |
| POP18 | 50 | coastal | N/A | Lewis R. | E. F. Lewis R. |
| POP19 | 50 | coastal | N/A | Lewis R. | N. F. Lewis R. |
| POP20 | 43 | coastal | N/A | Mill R. | Mill Cr. |
| POP21 | 28 | coastal | N/A | Sandy R. | Still Cr. |
| POP22 | 52 | coastal | N/A | Hood R. | East Fork Hood R. |
| POP23 | 35 | coastal | N/A | Hood R. | West Fork Hood R. |
| POP24 | 50 | coastal | N/A | White Salmon R. | Big White Salmon R. |
| POP27 | 45 | inland | KLICK | Klickitat R. | Lower Summit Cr. |
| POP30 | 47 | inland | KLICK | Klickitat R. | Snyder Cr. |
| POP33 | 46 | inland | KLICK | Klickitat R. | Upper Trout Cr. |
| POP35 | 50 | inland | MGILCS | Deschutes R. | Buckhollow Cr. |
| POP39 | 50 | inland | MGILCS | Deschutes R. | Trout Cr. |
| POP40 | 50 | inland | MGILCS | Fifteen R. | Fifteenmile Cr. |
| POP47 | 18 | inland | MGILCS | N. F. John Day R. | Granite Cr. |
| POP48 | 50 | inland | MGILCS | N. F. John Day R. | Middle N. F. J. D. |
| POP49 | 18 | inland | MGILCS | S. F. John Day R. | Deer Cr. |
| POP53 | 50 | inland | MGILCS | Umatilla R. | Iskuulpa Cr. |
| POP57 | 50 | inland | YAK | Yakima R. | Nile/ Naches R. |
| POP61 | 46 | inland | YAK | Yakima R. | Satus Cr. |
| POP63 | 50 | inland | UPCOL | Wenatchee R. | Chiwaukum R. |
| POP67 | 50 | inland | UPCOL | Wenatchee R. | Peshastin R. |
| POP69 | 50 | inland | UPCOL | Entiat R. | Entiat R. |
| POP70 | 50 | inland | UPCOL | Methow R. | Methow R. |
| POP74 | 50 | inland | MGILCS | lower Snake R. | Tucannon R. |
| POP76 | 50 | inland | MGILCS | lower Snake R. | Asotin Cr. |
| POP81 | 50 | inland | MGILCS | Lower Clearwater R. | Big Bear Cr. |
| POP82 | 50 | inland | MGILCS | Lower Clearwater R. | E. F. Potlatch R. |
| POP85 | 35 | inland | UPCLWR | M. F. Clearwater R. | Bear/ Selway R. |
| POP86 | 45 | inland | UPCLWR | M. F. Clearwater R. | Gedney/ Selway R. |
| POP90 | 47 | inland | UPCLWR | M. F. Clearwater R. | OHara/ Selway R. |
| POP94 | 38 | inland | UPCLWR | M. F. Clearwater R. | Colt/ Lochsa R. |
| POP95 | 44 | inland | UPCLWR | M. F. Clearwater R. | Crooked Fork/Lochsa R. |
| POP98 | 38 | inland | UPCLWR | M. F. Clearwater R. | Storm/ Lochsa R. |
| POP99 | 45 | inland | SFCLWR | S. F. Clearwater R. | Clear Cr. |
| POP101 | 36 | inland | SFCLWR | S. F. Clearwater R. | Johns Cr. |
| POP102 | 46 | inland | SFCLWR | S. F. Clearwater R. | Tenmile Cr. |
| POP107 | 45 | inland | MGILCS | Grande Ronde R. | Joseph Cr. |
| POP109 | 45 | inland | MGILCS | Grande Ronde R. | Lostine R. |
| POP110 | 50 | inland | MGILCS | Grande Ronde R. | Menatchee R. |
| POP111 | 50 | inland | MGILCS | Grande Ronde R. | Wenaha R. |
| POP112 | 50 | inland | MGILCS | Imnaha R. | Big Sheep Cr. |
| POP113 | 23 | inland | MGILCS | Imnaha R. | Camp Cr. |
| POP114 | 44 | inland | MGILCS | Imnaha R. | Cow Cr. |
| POP119 | 45 | inland | SFSALM | S. F. Salmon R. | East Fork S. F. Salmon R. |
| POP121 | 45 | inland | SFSALM | S. F. Salmon R. | Secesh R. |
| POP122 | 45 | inland | SFSALM | S. F. Salmon R. | Stolle Meadows |
| POP124 | 32 | inland | MFSALM | Bargamin R. | Bargamin Cr. |
| POP127 | 46 | inland | MFSALM | M. F. Salmon R. | Lower Big Cr. |
| POP128 | 50 | inland | MFSALM | M. F. Salmon R. | Marsh Cr. |
| POP131 | 42 | inland | MFSALM | M. F. Salmon R. | Sulphur Cr. |
| POP132 | 45 | inland | MFSALM | M. F. Salmon R. | Upper Big Cr. |
| POP135 | 50 | inland | UPSALM | Upper Salmon R. | N. F. Salmon R. |
| POP138 | 44 | inland | UPSALM | Upper Salmon R. | Valley Cr. |
| POP139 | 50 | inland | UPSALM | Upper Salmon R. | W. F. Yankee Fork R. |
| POP140 | 50 | inland | SFCLWR | Hatchery | Dworshak |
| POP144 | 50 | inland | MGILCS | Hatchery | Tucannon/Lyons Ferry |
| POP141 | 50 | inland | UPSALM | Hatchery | Oxbow/ Hells Canyon |
| POP142 | 50 | inland | UPSALM | Hatchery | Pahsimeroi |
| POP143 | 50 | inland | UPSALM | Hatchery | Sawtooth |
| POP145 | 50 | inland | MGILCS | Hatchery | Wallowa |
|  |  |  |  |  |  |

Table S2. Summary statistic from abundance surveys in Rock Creek (2008 – 2012). Samples were grouped by river reach. Size data was available for 5,573 of 6,218 survey samples. Age proportions were inferred from fork lengths: age-1+ >70mm in spring and >90mm in the fall. Smaller fish were designated age-0. Fish > 150mm FL were deemed age-2+ or “mature”. Significant differences in age structure within and between watersheds are shaded grey.

| statistic | |  | lower watershed (Rock Cr.) | | | | lower watershed (Squaw Cr.) | | |  |  | upper watershed | | |
| --- | --- | --- | --- | --- | --- | --- | --- | --- | --- | --- | --- | --- | --- | --- |
|  |  |  | rkm1-9 | rkm15-19 | rkm20-22 |  | rkm S13-15 | rkmS15-21 | rkmS21-22 | overall |  | Ekone | Quartz | overall |
|  |  |  |  |  |  |  |  |  |  |  |  |  |  |  |
| survey total (n) | |  | 397 | 1,282 | 380 |  | 1264 | 1,408 | 842 | *5,573* |  | 90 | 21 | *111* |
|  |  |  |  |  |  |  |  |  |  |  |  |  |  |  |
| **spring** | |  |  |  |  |  |  |  |  |  |  |  |  |  |
| (n) | age-0 |  | 111 | 170 | 5 |  | 513 | 333 | 121 | *1,253* |  | 3 | 0 | *3* |
|  | age-1+ |  | 8 | 73 | 57 |  | 117 | 172 | 217 | *644* |  | 19 | 0 | *19* |
|  | mature |  | 5 | 13 | 4 |  | 23 | 9 | 12 | *66* |  | 10 | 0 | *10* |
|  |  |  |  |  |  |  |  |  |  |  |  |  |  |  |
| (%) | age-0 |  | 0.90 | 0.66 | 0.08 |  | 0.79 | 0.65 | 0.35 | *0.64* |  | 0.09 | --- | *---* |
|  | age-1+ |  | 0.06 | 0.29 | 0.86 |  | 0.18 | 0.33 | 0.62 | *0.33* |  | 0.59 | --- | *---* |
|  | mature |  | 0.04 | 0.05 | 0.06 |  | 0.04 | 0.02 | 0.03 | *0.03* |  | 0.31 | --- | *---* |
|  |  |  |  |  |  |  |  |  |  |  |  |  |  |  |
| mean FL | age-0 |  | 46.6 | 48.1 | 38.0 |  | 49.0 | 48.0 | 49.4 | *48.4* |  | 33 | --- | *---* |
|  | age-1+ |  | 131.8 | 128.0 | 117.5 |  | 112.0 | 118.8 | 114.5 | *117.2* |  | 116.1 | --- | *---* |
|  | mature |  | 163.6 | 157.5 | 165.5 |  | 169.0 | 178.1 | 161.4 | *166.0* |  | 164.8 | --- | *---* |
|  |  |  |  |  |  |  |  |  |  |  |  |  |  |  |
| **fall** | |  |  |  |  |  |  |  |  |  |  |  |  |  |
| (n) | age-0 |  | 180 | 828 | 83 |  | 479 | 616 | 192 | *2,378* |  | 8 | 0 | *8* |
|  | age-1+ |  | 58 | 211 | 235 |  | 155 | 287 | 312 | *1,258* |  | 31 | 11 | *42* |
|  | mature |  | 35 | 131 | 38 |  | 57 | 138 | 35 | *434* |  | 19 | 10 | *29* |
|  |  |  |  |  |  |  |  |  |  |  |  |  |  |  |
| (%) | age-0 |  | 0.66 | 0.71 | 0.23 |  | 0.69 | 0.59 | 0.36 | *0.58* |  | 0.14 | --- | *0.10* |
|  | age-1+ |  | 0.21 | 0.18 | 0.66 |  | 0.22 | 0.28 | 0.58 | *0.31* |  | 0.53 | 0.52 | *0.53* |
|  | mature |  | 0.13 | 0.11 | 0.11 |  | 0.08 | 0.13 | 0.06 | *0.11* |  | 0.33 | 0.48 | *0.37* |
|  |  |  |  |  |  |  |  |  |  |  |  |  |  |  |
| mean FL | age-0 |  | 74.5 | 68.3 | 67.2 |  | 69.6 | 68.2 | 61.7 | *68.4* |  | 67.0 | --- | --- |
|  | age-1+ |  | 106.6 | 126.8 | 124.0 |  | 126.4 | 126.1 | 121.5 | *123.8* |  | 118.3 | 118.3 | *118.3* |
|  | mature |  | 175.4 | 166.3 | 164.4 |  | 164.3 | 174.6 | 164.9 | *169.1* |  | 171.1 | 168.5 | *170.2* |
|  |  |  |  |  |  |  |  |  |  |  |  |  |  |  |

Table S3. Origins of 37 adult steelhead detected via PIT-tag transceiver in Rock Creek. Fish were tagged at Bonneville Dam as adults, all others were tagged as juveniles; hatchery-origin (H) and natural-origin (N). The farthest upsteam PIT-tag detection site of adult fish that eventually swam into Rock Creek is: RC=Rock Creek, MCN=McNary Dam fishway, and ICH=Ice Harbor Dam. Assignment likelihood scores LS>0.75 are bolded. Origins of 26 steelhead were known from release site information (*). The number (n>1) of detections of unique steelhead from each mark/release site appears in parentheses. See supplemental Table1 for reference ID.

| adult PIT-tag detections | |  | farthest upstream detection | | | |  | | GSI assigned origin | |
| --- | --- | --- | --- | --- | --- | --- | --- | --- | --- | --- |
| mark site | release site |  | RC | MCN | ICH |  | | reference | | *p* |
|  |  |  |  |  |  |  | |  | |  |
| Bonneville | Columbia R. |  | - | - | H |  | | MGILCS | | 0.722 |
| Bonneville | Columbia R. |  | - | N | - |  | | Rock Cr. | | **0.936** |
| Bonneville | Columbia R. |  | - | N | - |  | | SFCLWR | | 0.392 |
| Bonneville | Columbia R. |  | - | N | - |  | | UPSALM | | **0.929** |
| Bonneville | Columbia R. |  | - | N | - |  | | UPCOL | | **0.955** |
| Bonneville | Columbia R. |  | - | N | - |  | | UPCOL | | 0.580 |
| Bonneville | Columbia R. |  | N | - | - |  | | Rock Cr. | | **0.971** |
| Bonneville | Columbia R. |  | N | - | - |  | | UPSALM | | **0.804** |
| Bonneville | Columbia R. |  | N | - | - |  | | UPSALM | | 0.351 |
| Bonneville | Columbia R. |  | N | - | - |  | | MGILCS | | 0.492 |
| Bonneville | Columbia R. |  | N | - | - |  | | UPSALM | | **0.814** |
|  |  |  |  |  |  |  | |  | |  |
| Trout Cr. | Deschutes R. |  | - | H | - |  | | *MGILCS | | - |
| Lower Granite Dam | barged |  | N (3) | N (5); H (2) | N (2) |  | | *MGILCS | | - |
| Irrigon Hatchery | Imnaha R. |  | - | H | - |  | | *MGILCS | | - |
| Hagerman-NFH | upper Salmon R. | | - | H | - |  | | *UPSALM | | - |
| Clearwater Hatchery | Clearwater R. |  | - | H | - |  | | *SFCLWR | | - |
| Magic Valley Hatchery | Salmon R. |  | - | - | H (2) |  | | *UPSALM | | - |
| Imnaha R. | Imnaha R. |  | - | H | - |  | | *MGILCS | | - |
| Rock Creek | Rock Creek |  | N (6); H (1) | - | - |  | | *Rock Creek | | - |
|  |  |  |  |  |  |  | |  | |  |
